# Associations between glycated albumin and current measures of glycaemic control in Saudi adults

**DOI:** 10.3389/fendo.2025.1731945

**Published:** 2026-01-19

**Authors:** Suhad Bahijri, Aliaa Sabban, Sumia Enani, Maha Saleh Alqahtani, Manal Malibary, Mohammad Alhashmi, Jaakko Tuomileto

**Affiliations:** 1 Department of Clinical Biochemistry, Faculty of Medicine, King Abdulaziz University, Jeddah, Saudi Arabia; 2 Saudi Diabetes Research Group, King Fahd Medical Research Centre, King Abdulaziz University, Jeddah, Saudi Arabia; 3 Food, Nutrition and Lifestyle Research Unit, King Fahd for Medical Research Centre, King Abdulaziz University, Jeddah, Saudi Arabia; 4 Department of Food and Nutrition, Faculty of Human Sciences and Design, King Abdulaziz University, Jeddah, Saudi Arabia; 5 Department of Medical Laboratory Sciences, Faculty of Applied Medical Sciences, King Abdulaziz University, Jeddah, Saudi Arabia; 6 Toxicology and Forensic Sciences Unit, King Fahd Medical Research Centre, King Abdulaziz University, Jeddah, Saudi Arabia; 7 Department of Public Health, University of Helsinki, Helsinki, Finland; 8 Department of Public Health and Welfare, Finnish Institute for Health and Welfare, Helsinki, Finland

**Keywords:** biomarkers/blood, diabetes mellitus/diagnosis, glycated albumin, hyperglycaemia/diagnosis, ROC curve

## Abstract

**Background:**

Current dysglycaemia detection methods have limits; glycated albumin (GA), unaffected by conditions that distort HbA1c, is proposed as an alternative. We aimed to estimate the relationship between various glycaemic parameters and their association with GA in Saudi adults to evaluate GA potential utility in screening, detecting, and monitoring diabetes (DM) and intermediate hyperglycaemia (IH).

**Method:**

A total of 132 biobank serum samples (-80°C) representing a wide glycaemia range, using HbA1c, fasting plasma glucose -FPG, and 1 hour plasma glucose- 1h-PG data. Serum GA was measured by ELISA and expressed as %. Correlations with glycaemic markers were assessed, group means (normoglycaemia, IH, DM) were compared, and diagnostic performance evaluated by ROC analysis. Optimal GA cut-offs for dysglycaemia and DM were determined, with significance set at P< 0.05.

**Results:**

Used measures of glycaemia did not consistently classify glycaemic status in the same way. The groups with IH and DM had significantly higher mean GA values compared with the normoglycaemia group (P<0.001). GA values correlated significantly with all glycaemic markers (P<0.001), showing the strongest correlation with HbA1c, and the weakest with 1h-PG. the optimal GA cut-off values for detecting dysglycaemia was 13.9% (Sensitivity= 0.786, specificity= 0.917), and 14.7% (Sensitivity= 0.857, specificity= 0.747) for DM.

**Conclusion:**

GA correlated significantly with other markers and can be suggested as an alternative to detect and monitor glycaemic status among Saudis. Further research is required to determine ranges in our population.

## Introduction

The prevalence of dysglycaemia and its components, diabetes mellitus (DM) and intermediate hyperglycaemia (IH- also called prediabetes) - including impaired fasting glucose (IFG) and impaired glucose tolerance (IGT) - are increasing globally (1, 2). DM and dysglycaemia, disorders of glucose homeostasis of different degrees, which, if left undiagnosed and unmanaged, can result in damage to multiple organs (1, 2). Therefore, early detection of all forms of disorders in glucose homeostasis is utmost important to prevent their potential deleterious effects.

DM and IH have been traditionally diagnosed using fasting plasma glucose (FPG) or 2-h plasma glucose (2-h PG) after a 75-g oral glucose tolerance test (OGTT) (3). However, the association between FPG and 2-h PG is affected by various factors resulting in a significant variability in the classification of glycaemic status (4). Both measurements have limitations and disadvantages; the optimal FPG cut-off value for diagnosing is less sensitive than the 2-hPG (5–9), while the 2-h OGTT is time consuming (10). The American Diabetes Association (ADA) endorsed the use of glycohemoglobin (HbA1c) as a diagnostic criterion for DM in 2010 (11) based on the recommendation of the International Expert Committee (IEC) (12). However, HbA1c also has many disadvantages. It is unreliable in many physiological and clinical conditions due to factors that affect lifespan of the erythrocytes and haemoglobin variants. In addition, HbA1c is a less sensitive biomarker for detecting dysglycaemia than glucose measurement (13–15).

To achieve the best possible sensitivity and specificity it was suggested to use a combination method (HbA1c and FPG) for diabetes screening and diagnosis using HbA1c not as an alternative but as an adjunct to FPG (16, 17). The practicability and cost effectiveness of this remains to be evaluated.

Interest in the 1-h post-challenge PG has re-emerged in the early 2000s (18). Studies have confirmed its association with insulin sensitivity and pancreatic β-cell function (19–21). In addition, several studies across diverse populations have reported that the 1-h PG is superior to FPG, 2-h PG, and HbA1c in predicting incident DM (19, 22–25), and various complications associated with DM, including cardiovascular disease (26) in individuals with normal glucose tolerance (NGT) with current criteria. A petition was published proposing the use of 1-hPG for diagnosing IGT, suggesting a cut-off point of 8.6 mmol/l (27). Recently, a Position Statement by the International Diabetes Federation was published recommending that people with a 1-h PG ≥ 155 mg/dL (8.6 mmol/L) are considered to have IH, while those with a 1-h PG ≥ 209 mg/dL (11.6 mmol/L) are classified as having DM (28).

To avoid glucose loading and the need for fasting, another glycated protein with a shorter half-life than haemoglobin, namely glycated albumin (GA), was proposed as a biomarker of glycaemic status (29). Albumin, which is produced in the liver, is considered the major protein in blood, comprising about 60% of serum proteins (30). It has many important physiological roles including maintenance of colloid-osmotic pressure, acting as a carrier to various hormones, metal ions, fatty acids and bilirubin, as well as having anti-oxidant activities (30). In addition, albumin level has been long used in the assessment of nutritional status with low levels indicating malnutrition (31). Low Albumin level has also been associated with inflammation (32), and various other conditions including liver disease, kidney disease, acute pulmonary embolism and cancer (33, 34). Albumin in plasma is glycated by a non-enzymatical reaction faster than haemoglobin (35). Therefore, it may reflect changes in glycaemic status earlier than HbA1c, and detect any fluctuations following medical therapy faster due to the shorter life of albumin (36). Moreover, unlike HbA1C, GA level is not affected by the presence of various hemoglobinopathies (37) anaemia, or pregnancy (38). Indeed, results of various clinical studies on different populations and age groups have indicated that GA is a promising marker in DM (39–42). Moreover, GA was reported to be better than the HbA1c for evaluating short-term changes in plasma glucose and hence may be considered as a suitable measure for the effectiveness of anti-diabetic medication in type 2 diabetic patients (43). Another study found linear associations between serum GA, plasma glucose, and HbA1c, and in cases where HbA1c did not adequately reflect the glycaemic status in the diagnosis of diabetes, serum GA provided a valuable substitute especially that it is more practical and saves more time than performing an OGTT (44). In addition, several studies have indicated that in patients with diabetes and chronic kidney disease, GA was found to be a better marker of glycaemic control (45–47), especially that HbA1C is not a reliable marker in these cases (47). Furthermore, studies reported an association of GA with the chronic complications of type 1 (48) and type 2 diabetes (49).

In view of the high prevalence and incidence of diabetes in Saudi Arabia (50) there is an increased need for an effective test to screen, detect, and monitoring DM and IH among Saudis. Apart of a small study to evaluate the association between glycated-albumin and various biochemical parameters in long-standing (>10 years) type-2 diabetic subjects (51), no studies evaluating the value of GA measurements as a marker of glycaemic control among the Saudi adults with a wide range of glycaemic status have been carried out.

Therefore, the aim of this study was to estimate the association between various glycaemic parameters in Saudi adults - including HbA1c, FPG, and 1h-PG and their association with GA to evaluate its potential utility in screening, detecting, and monitoring DM and IH.

## Materials and methods

### Study design and population

A cross-sectional design was adopted for this study. Ethical approval was obtained from the Committee on Ethics of Human Research at the Faculty of Medicine, King Abdulaziz University (Approval No. 345-22, dated June 30, 2022). All procedures involving human participants and use of stored biological samples were conducted in accordance with the ethical standards of the institutional and/or national research committee and with the 1964 Declaration of Helsinki and its later amendments or comparable ethical standards.

A total of 132 serum samples from the biobank stored at -80°C in the Food, Nutrition and Lifestyle Research Unit at King Fahad Medical Research Centre (KFMRC) and collected earlier from different health care centres all over the city of Jeddah as outlined in an earlier study (52) to ensure socioeconomic and ethnic diversity were chosen to represent a wide range of glycaemia, based on HbA1c, FPG, and 1h-PG. In summary, a cross-sectional design was used aiming to recruit 1500 (750 men and 750 women) participants from selected public healthcare centres by employing stratified, 2-stage cluster sampling method (53). A consent form was signed by recruited participants. Demographics, lifestyle variables, dietary habits, and personal medical and family history were obtained using a predesigned questionnaire based on factors associated with dysglycaemia found in other populations (54–57).

Participants were instructed to fast overnight for 8 to 14 hours, and fasting blood sample was collected to estimate fasting plasma glucose (FPG) and glycated haemoglobin (HbA1c). Another sample was collected 1 hour after ingestion of 75-g glucose solution (CASCO NERL Diagnostics, East Providence, RI, USA) for estimating plasma glucose (1-hour oral glucose tolerance test; 1 h-OGTT) (58, 59). All samples (whole blood or plasma) were analysed at the Clinical Chemistry Laboratory at National Guard Hospital in Jeddah. HbA1c was measured with HbA1c analyser G8 (TOSOH Corporation, Japan). Plasma glucose was measured by spectrophotometric methods using Architect c8000 autoanalyzer (ABBOTT, USA).

The individuals were classified into three categories: (i) those within normal glycaemic range (normoglycaemia), (ii) those within the intermediate glycaemic range (IH), and (iii) those within the diabetic range (DM) using a combined glycaemic reference, in which, placement into a range was assigned if any of the three measures (HbA1c, FPG or 1-h PG) fell within the corresponding range (28, 60). All samples were collected from volunteers without prior identification of dysglycaemia. Participants samples with HbA1c concentrations of 5.7-6.4%, and/or FPG of 5.6-6.9 mmol/L, and/or 1h-PG of 8.6-11.5 mmol/L were classified as IH, while participants with HbA1c ≥6.5%, or FPG ≥ 7 mmol/L, or 1h-PG ≥11.6 mmol/L were classifies as having DM. Dysglycaemia was defined as the presence of either IH or DM. Participants with any acute or chronic conditions that may significantly impact blood albumin or glucose metabolism including hemoglobinopathies, kidney and liver diseases were excluded.

As a result, 52 samples reflected DM status, 32 reflected IH, and 48 reflected normoglycaemia.

Demographic and anthropometric data, including age, sex, and BMI, were retrieved from the participants records stored in the unit files. Similarly, results of the medical investigation, including the biochemistry profile - comprising FPG, HbA1c,1h-PG, total serum albumin, and lipid profile - were also collected from the records.

### Biochemical assay for GA

GA was measured in samples using an ELISA kit (CUSABIO GA kit, Cusabio Technology LLC, China) to quantitatively determine human GA concentrations in serum and plasma and following the manufacturer’s instructions. The assay is based on competitive inhibition reaction. A microtiter plate, pre-coated with GA, is provided in the kit. standards or samples are added into the designated wells along with a Horseradish Peroxidase (HRP) conjugated antibody specific to GA. A competitive binding interaction occurs between the immobilised GA and GA present in the samples. After incubation, a substrate solution is added, leading to a colorimetric reaction that is inversely proportional to the GA concentration in the samples. Absorbance was measured using a microplate reader (DNM-9602, Drawell International Technology Limited Co., China). The percentage of GA was calculated using the formula: (GA%= serum GA/total albumin x100) (61). Quality assurance was maintained by random incorporation of standard reference materials into the sample plates, ensuring the results consistently met satisfactory criteria.

### Statistical analysis

All statistical analyses were performed using g IBM SPSS statistics version 20.0 for Windows (IBM Corporation, Armonk, NY, USA). Data were analysed and descriptive statistics were expressed as frequencies or mean ± standard deviation. Variables with homogeneous variances were compared across glycaemic groups using one-way ANOVA with Bonferroni *post-hoc* tests, whereas variables with heterogeneous variances were compared using Welch’s ANOVA with Games-Howell *post-hoc* comparisons. Correlation between markers of glycaemic status (FPG, 1-hPG, and HbA1c) and GA were assessed by the Spearman’s test. The ANOVA test was used to compare normoglycaemic, IH, and DM groups.

The performance of serum GA in identifying dysglycaemia (defined as IH or DM), and specifically DM – based on the three measures of glycaemic status (HbA1c, FPG, and 1h-PG) – was evaluated using the receiver operating characteristic (ROC) curve. As participants were classified into normoglycaemia, IH and DM using a combined glycaemic reference in which placement into a range was assigned if any of the three measures fell within the corresponding range, the combined definition was used as the reference “true status” in the ROC analysis. The optimal cut-off for serum GA was derived from the ROC curve with the shortest distance to the top-left corner in the ROC curve and the Youden index (Y = sensitivity + specificity–1). The diagnostic performance of various GA thresholds (ranging from GA of 10.0% to 23.0%, at 0.1% intervals) for identifying dysglycaemia and DM was calculated, including the sensitivity, specificity, positive predictive value (PPV), negative predictive value (NPV), false negative rates (FNR), and false positive rates (FPR). A p-value of<0.05 was considered to be statistically significant.

## Results

### Characteristics of the study participants

Data for all variables were available for all 132 individuals except for 1h-PG. 1h-PG values were available for 96 individuals only. Characteristics of the study participants grouped according to their glycaemic status, are outlined in **Table 1**. Across the three glycaemic groups, significant differences were observed in several biochemical and clinical variables. HbA1c, fasting glucose, 1-h glucose, and glycated albumin were progressively higher from normoglycaemia to intermediate hyperglycaemia and from intermediate hyperglycaemia to diabetes (all p< 0.001), and pairwise comparisons were significant for all three group contrasts. Triglycaerides was also elevated in the diabetes group compared with normoglycaemia (p< 0.01) and albumin was lower in the diabetes group compared with normoglycaemia (p< 0.01). Age, BMI, systolic blood pressure, and diastolic blood pressure differed significantly among groups, with higher values observed in the diabetes group compared with normoglycaemia (all p ≤ 0.03). Total cholesterol, LDL-c, HDL-c, height, and weight did not differ significantly between groups (all p > 0.05).

**Table 1:** Baseline characteristics of study participants by glycaemic status.

Variables	Normoglycaemia (n =48)	IH (n = 32)	DM (n = 52)	P-value
Sex, n (%)^				
Men	24 (32%)	20 (26.7%)	31 (41.3%)	0.473
Women	24 (42.1%)	12 (21.1%)	21 (36.8%)	
Age (yr)^†^	31.7 ± 13.9	47 ± 15.4	44.1 ± 12.2	<0.001
Weight (kg)^‡^	79.9 ± 24.2	82.1 ± 15.9	86.8 ± 19.5	0.254
Height (cm)^†^	165.8 ± 9.8	165.6 ± 9.1	163 ± 10.3	0.317
BMI (kg/m²)^†^	29.1 ± 7.8	30.8 ± 5.5	32.9 ± 7.7	0.035
SBP (mmHg)^†^	114.9 ± 14.2	123 ± 13.1	127.4 ± 21.4	0.002
DBP (mmHg)^†^	69.3 ± 12	76 ± 11.6	75.6 ± 11	0.010
HbA1c (%)^‡^	5.1 ± 0.3	5.9 ± 0.2	8.4 ± 1.9	<0.001
FPG (mmol/L)^‡^	4.2 ± 0.5	4.9 ± 0.6	8.9 ± 3.7	<0.001
1h-PG (mmol/L)^‡^	5.9 ± 1.4	9.8 ± 0.8	13.3 ± 3.8	<0.001
TC (mmol/L)^†^	4.7 ± 0.9	5.1 ± 1.2	4.9 ± 1.3	0.207
TG (mmol/L)^‡^	1.1 ± 0.5	1.6 ± 0.8	2 ± 1.5	<0.001
LDL-c (mmol/L)^†^	3.2 ± 0.8	3.4 ± 1	2.9 ± 1.1	0.096
HDL-c (mmol/L)^†^	1.3 ± 0.3	1.2 ± 0.2	1.2 ± 0.3	0.075
GA (%)^‡^	12.1 ± 2.4	14.1 ± 2.8	18.1 ± 4.3	<0.001
Serum albumin (g/L)	42.7 ± 1.8	42.8 ± 1.9	40.8 ± 2.7	<0.001

Data are presented as mean ± SD or number (percentage). Sex distribution was analysed by chi-square test (^^^). Continuous variables were analysed using one-way ANOVA (^†^) when variances were homogeneous or Welch’s ANOVA (^‡^) when variances were heterogeneous. P-values indicate overall group differences.

IH, intermediate glycaemic range; DM, diabetic range; BMI, body mass index; SBP, systolic blood pressure; DBP, diastolic blood pressure; HbA1c, glycated haemoglobin; FPG, fasting plasma glucose; 1h-PG, 1-hour plasma glucose; TC, total cholesterol; TG, triglycaerides; HDL-c, high-density lipoprotein cholesterol; LDL-c, low-density lipoprotein cholesterol; GA, glycated albumin.

### Correlation between GA with the other glycaemic measurements

A highly significant Spearman correlation coefficient was observed between GA and all glycaemic markers, as presented in **Table 2**.

**Table 2:** Correlation of GA with other glycaemic markers.

Variable		HbA1c	FPG	1h-PG
HbA1c	R	1		
	P-value	.		
FPG	R	0.831	1	
	P-value	<0.001	.	
1h-PG	R	0.865	0.758	1
	P-value	<0.001	<0.001	.
GA	R	0.686	0.638	0.487
	P-value	<0.001	<0.001	<0.001

Spearman correlation coefficients (R) and corresponding p-values are shown for relationships between GA and HbA1c, fasting plasma glucose (FPG), and 1-hour post-load glucose (1h-PG). HbA1c, glycated haemoglobin A1c; FPG, fasting plasma glucose; 1h-PG, one hour plasma glucose; GA, glycated albumin.

**Figure 1:**
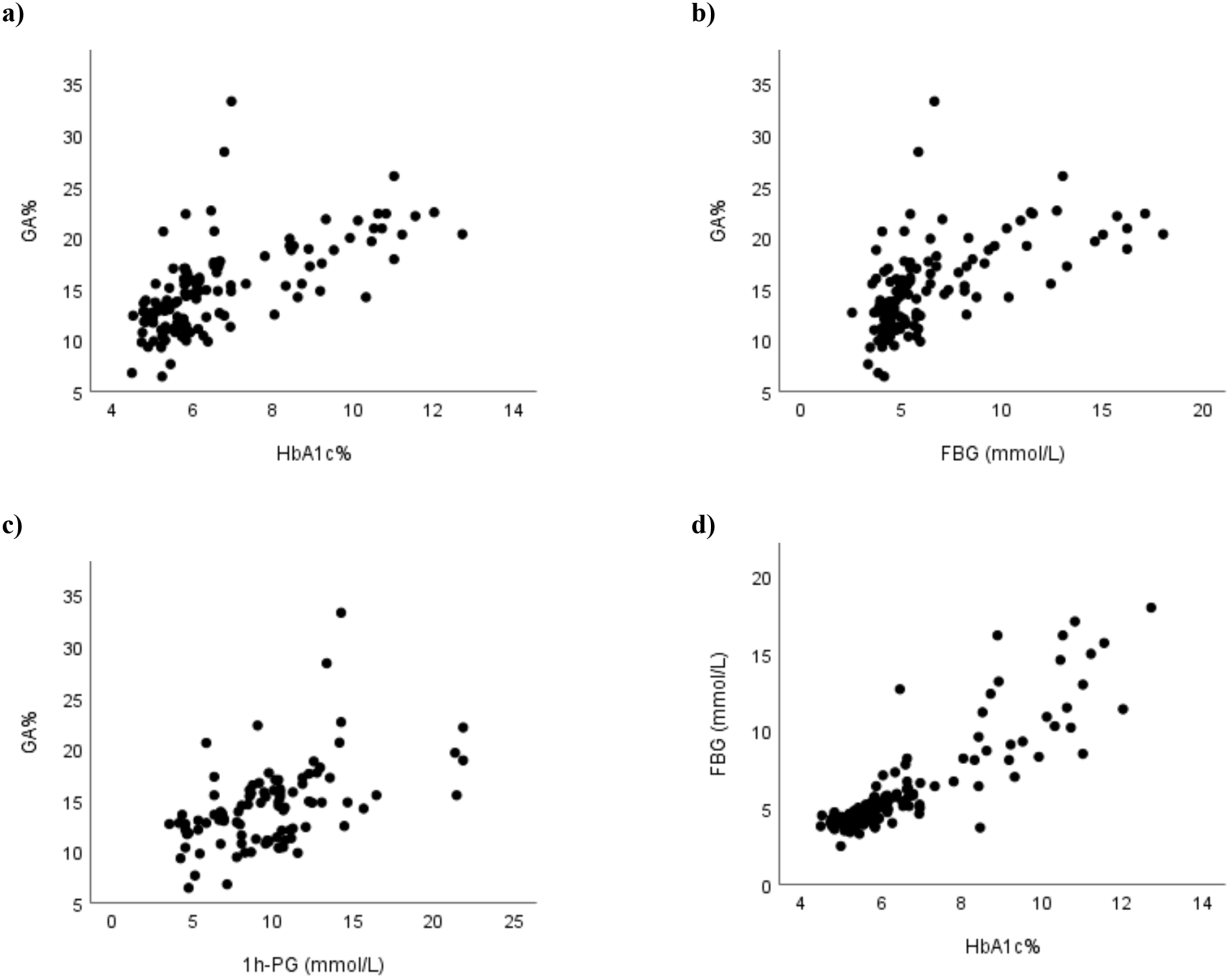
Correlations between glycaemic measures **(a–d)**: **(a)** HbA1c vs GA, **(b)** FPG vs GA, **(c)** 1h-PG vs GA, and **(d)** HbA1c vs FPG. HbA1c, glycated haemoglobin A1c; FPG, fasting plasma glucose; 1h-PG, 1-hour plasma glucose; GA, glycated albumin.

The strongest correlation coefficient with GA was observed with HbA1c, while the weakest with 1h-PG. HbA1c also showed significant correlations with both FPG and 1h-PG to a similar extent. Additionally, a strong and significant correlation was found between FPG and 1h-PG (r= 0.758). **Figure 1** displays the relationships between GA and HbA1c (**Figure 1a**), GA and FPG (**Figure 1b**), GA and 1-hPG and (**Figure 1c**), GA and (**Figure 1d**), and FPG and HbA1c (**Figure 1d**).

### The proportion of people identified with hyperglycaemia (IH and diabetes) through HbA1c, FPG and 1h-PG

Different measures of hyperglycaemia classified the samples differently. Based on HbA1c measurements, 36.4% (n=48) were normoglycaemic, 31.8% (n=42) were classified as having IH, and 31.8% (n=42) DM. According to FPG results, 71.2% (n=94) were normoglycaemic, 6.8% (n=9) were classified as having IH, and 22% (n=29) DM. Using 1h-PG values, 28.8% (n=38) were normoglycaemic, 23.5% (n=31) had IH, and 20.5% (n=27) had DM.

**Figure 2:**
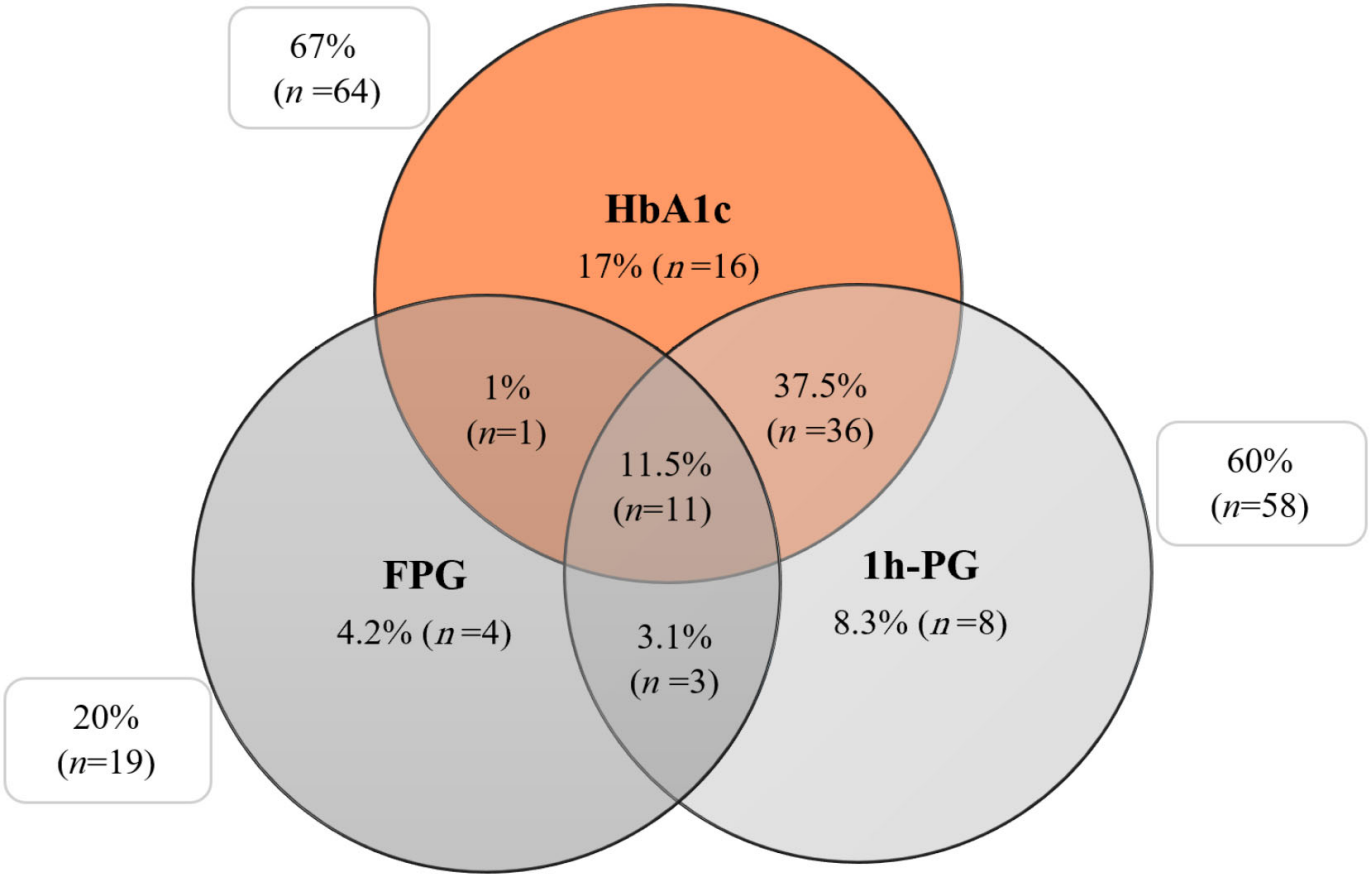
The percentage of individuals detected as having dysglycaemia through HbA1c, FPG, and 1h-PG. HbA1c, glycated haemoglobin A1c; FPG, fasting plasma glucose; 1h-PG, 1-hour plasma glucose.

A total of 96 samples were tested for all three measures - HbA1c, FPG and 1h-PG. Although significant correlations were observed between HbA1c with both FPG and 1h-PG, the three measures did not consistently classify glycaemic in the same way. The concordance between the three measures in classifying of glycaemic status is illustrated in the Venn diagram (**Figure 2**).

Based on HbA1c, 67% (n=64) of the samples showed dysglycaemia (IH or DM), with 17% (n=16) identified by HbA1c alone. Similarly, 60% (n=58) were classified as having dysglycaemia using 1h-PG, with 8.3% (n=8) detected exclusively by 1h-PG alone. Additionally, 20% (n=19) of the samples showed dysglycaemia based on FPG, with 4.2% (n=4) identified only through FPG analysis. Only 11.5% (n=11) of all dysglycaemic samples showed elevated values across all three measures of glycaemia simultaneously.

### Prevalence of dysglycaemia at different levels of glycated albumin

The prevalence of dysglycaemia at different levels of GA is presented in **Table 3**.

**Table 3:** Prevalence of dysglycaemia, IH and DM across GA categories.

GA (%)	n	Dysglycaemia n (%)	IH n (%)	DM n (%)
<14	62	18 (29%)	12 (19%)	6 (10%)
14–14.9	13	12 (100%)	6 (46%)	7 (54%)
15–15.9	14	12 (86%)	7 (50%)	5 (36%)
16–16.9	5	5 (100%)	4 (80%)	1 (20%)
17–17.9	12	11 (92%)	2 (17%)	9 (75%)
18–18.9	4	4 (100%)	0 (0%)	4 (100%)
19–19.9	5	5 (100%)	0 (0%)	5 (100%)
≥20	17	16 (94%)	1 (6%)	15 (88%)

Data are presented as numbers and percentages within each glycated albumin (GA) category.

Participants were classified into normal glycaemic range (normoglycaemia), intermediate glycaemic range (IH), diabetic range (DM) using a combined glycaemic reference, in which, placement into a range was assigned if any of the three measures (glycated haemoglobin (HbA1c), fasting plasma glucose (FPG) and 1-hour plasma glucose (1h-PG)) fell within the corresponding range. Dysglycaemia includes IH and DM combined. The combined definition was used as the reference “true status” in the ROC analysis.

### Diagnostic performance of glycated albumin for identifying dysglycemia (IH or DM) and DM

**Figure 3:**
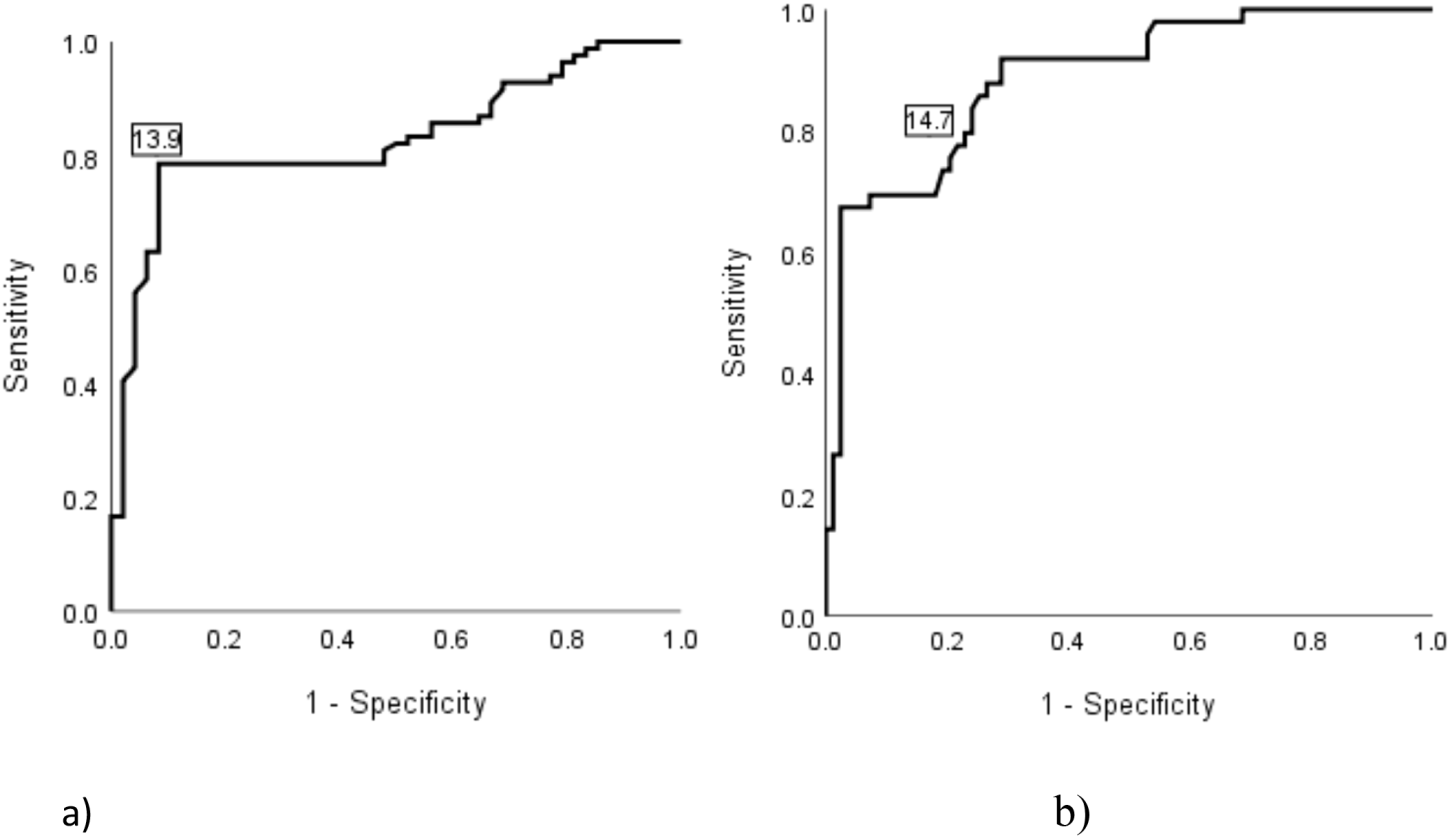
The discriminative power of glycated albumin % for detecting dysglycaemia (diabetes or intermediate hyperglycaemia) **(a)** and DM alone **(b)**. Participants were classified into normal glycaemic range (normoglycaemia), intermediate glycaemic range (IH), diabetic range (DM) using a combined glycaemic reference, in which, placement into a range was assigned if any of the three measures (glycated hemoglobin (HbA1c), fasting plasma glucose (FPG) and 1-hour plasma glucose (1h-PG)) fell within the corresponding range. Dysglycaemia includes IH and DM combined. The combined definition was used as the reference “true status” in the ROC analysis. Panel **(a)** shows the ROC curve for detecting dysglycaemia (including diabetes and intermediate hyperglycaemia), with an optimal cut-off point of 13.9%. Panel **(b)** displays the ROC curve for detecting DM alone, with an optimal cut-off of 14.7%. Sensitivity is plotted against 1-specificity to evaluate diagnostic performance.

A GA level of 14% can be suggested as a cut-off point for detecting dysglycaemia in Saudi adults. To support this suggestion a ROC curve was constructed (**Figure 3**) to evaluate the discriminative power of GA in identifying metabolic abnormalities (IH or DM), as well as DM alone. The area under the curve (AUC), most appropriate cut-off point, as well as specificity and sensitivity were calculated for both conditions and presented in **Table 4**.

**Table 4:** Diagnostic performance of glycated albumin (%) for detecting dysglycaemia and DM.

	Glycated albumin							
	AUC (95% CI)	Optimal cut-off point	Sensitivity	Specificity	PPV	NPV	PLR	NLR
Dysglycaemia	**0.829 (0.758 – 0.9)**	**13.9**	0.786	0.917	0.943	0.710	9.429	0.234
Diabetes	**0.888 (0.83 – 0.945)**	**14.7**	0.857	0.747	0.667	0.899	3.388	0.191

Area under the curve (AUC) and its 95% CI, optimal cut-off point for glycated albumin % for detecting dysglycaemia (diabetes or intermediate hyperglycaemia) and DM alone.

Participants were classified into normal glycaemic range (normoglycaemia), intermediate glycaemic range (IH), diabetic range (DM) using a combined glycaemic reference, in which, placement into a range was assigned if any of the three measures (glycated hemoglobin (HbA1c), fasting plasma glucose (FPG) and 1-hour plasma glucose (1h-PG)) fell within the corresponding range. Dysglycaemia includes IH and DM combined. The combined definition was used as the reference “true status” in the ROC analysis.

Cl, 95% confidence intervals; PPV, positive predictive value; NPV, negative predictive value; PLR, positive likelihood ratio; and NLR, negative likelihood ratio. Bold values indicate statistically significant diagnostic performance metrics.

## Discussion

With the increasing prevalence of diabetes in Saudi Arabia (50), alongside the high rates of the medical conditions that limit the reliability of HbA1c as a glycaemic indicator (62–66) in the country, there is a growing need to identify an alternative marker of glycaemic status that is more reliable, cost-effective, time-efficient, and does not require fasting (67). Therefore, the aim of this pilot study was to examine how glycated albumin relates to other glycaemic control measurements (namely HbA1c, FPG, and 1h-PG) in order to evaluate its potential usefulness for screening, detection, and monitoring dysglycaemia and diabetes in the Saudi population.

The findings from this study clearly indicate that the currently recommended measures of glycaemia (HbA1c, FPG and 1h-PG) do not consistently classify glycaemic status of the samples in the same manner. Notable, only 11.5% of the samples identified dysglycaemia by all the three measures, highlighting the need for evaluating another, more robust and reliable biomarker of glycaemic status.

In this study, GA was measured and its correlations with HbA1c, FPG, and 1h-PG were investigated using the stored data of 132 serum samples collected from participants without prior diagnosis of disorders of glucose metabolism. The samples represented a broad range of glycaemic status - normoglycaemic, IH, and DM - Based on the currently recommended diagnostic criteria (28, 60). We found that people with IH and DM had significantly higher mean GA levels compared with those with normoglycaemia (P<0.001). Additionally, the mean GA values observed in our study were very similar to those reported in a comparable population sample from China (68) even though their sample size was 1935 subjects, which is much larger than our sample in this pilot study. Other studies from different countries gave similar results also. Large epidemiological cohorts have evaluated GA alongside other nontraditional glycaemic markers at substantially greater scale (e.g., ARIC analyses reporting GA in >10,000 adults) (69). providing narrower confidence intervals and greater power for subgroup analyses than is possible here. Conversely, several diagnostic cut-off studies have used intermediate sample sizes (hundreds to ~1,000); for instance, one Korean ROC analysis referenced an evaluation in 852 individuals (70). Using a different method for estimating GA than ours, and much smaller sample of 32 normoglycemic Italian adults, Paroni et al. estimated the reference interval for GA to be 11.7%-16.9% (71).Taken together, our sample size is smaller than many population cohorts, but it intentionally spans a wide glycaemic range and uses a combined glycaemic reference (HbA1c, FPG and 1h-PG) to strengthen classification; therefore, our findings are best interpreted as a Saudi-specific pilot estimate of GA discrimination that warrants confirmation in a larger, fully phenotyped cohort.

However, we were unable to compare our results for newly identified people with DM with previously published GA levels in a small study on Saudi people with long-standing T2DM (51) Differences between GA levels reported here and those previously published may reflect both biological/clinical differences in the sampled populations and methodological differences in GA measurement. The GA quantification in this previous study used a different commercial immunoassay kit with GA measured from a standard curve as a concentration readout on antigen-precoated plates, whereas the current manuscript used the CUSABIO competitive ELISA (competitive inhibition format) and then expressed GA as GA% (GA/total albumin ×100), which explicitly normalises to albumin concentration and may reduce between-person variability attributable to serum albumin differences (51). In addition, comparison was not appropriate for the following reasons: a) Enrolled subjects had long-standing T2DM, and must have been on various medications, unlike those in our study, b)Stated demographic and biochemical characteristics of enrolled subjects were different to the characteristics in our study, c) No coexisting complications were mentioned, but are expected in view of the reported HbA1c levels and the duration of DM, all of which could affect GA levels.

We also found that GA correlated significantly and strongly with all three measures of glycaemia. The strongest correlation was observed with HbA1c (r=0.701), while the weakest one was with 1h-PG. This pattern of correlations – highest with HbA1c, followed by FPG, and lowest 1-hPG is similar to that reported in a previous study conducted in China (68). Our findings are also in keeping with findings reported in previous studies in other populations on people with diabetes (39, 40, 42). The lower correlation coefficient between GA and FPG compared with that with HbA1c might be attributed to the nature of FPG as a point-in-time measure which can be affected by the duration of fasting and recent food intake. In contrast, both HbA1c like GA reflect average blood glucose levels over a period of several weeks, which may explain their stronger inter-correlation. The relatively weaker correlation between GA and 1h-PG might be partially explained by the smaller number of samples available for 1-hPG analysis. However, a more probable explanation is that the 1-hPG level, like FPG, is a point-in-time measure that depends on the shape of the glucose tolerance curve, which, in turn, depends on whether the studied individuals had impaired fasting glucose (IFG) or impaired glucose tolerance (IGT) (72). People having IFG show a fast increase in plasma glucose concentration with a peak at 1 hour, and a return to normal or near normal values after 2 hours, while people with IGT have a more gradual initial increase in plasma glucose concentration which continues to rise after 1 hour and remains markedly increased at 2 hours (72). In addition, people with NGT commonly present with a biphasic OGTT, while people with IGT usually have a monophasic OGTT (73, 74). Our sample included people with NGT as well as IFG, IGT and even some with diabetes, explaining the slightly lower correlation coefficient compared to GA which reflects average blood glucose levels over the previous weeks.

Nevertheless, the significant correlations observed across all three measures of glycaemia with GA support the potential utility of GA as a biomarker for detecting IH and DM among Saudi adults.

In this study, we identified the optimal GA cut-off values for detecting dysglycaemia and its components - IH and DM- with an excellent AUC, excellent specificity, and very good sensitivity. Previous studies conducted in various populations have reported a range of GA cut-off values (39, 44, 68, 75–77). For example, studies on Asian populations for diagnosing DM, have suggested optimal GA cut-off values between 14%-16%, whereas a lower cut-off value of 13.5% has been proposed for Caucasian populations (75).The observed variations in GA cut-off values across populations are likely attributed to study designs, differences in participant selection, laboratory methods, and environmental and genetic factors, highlighting the need to find out whether true population-based differences in GA values exist or whether uniform cut-off points should be adopted, similar to plasma glucose and HbA1c.

To enhance the diagnostic power of GA for diagnosing or excluding DM, it was suggested to use FPG and GA in combination (44). While we did not attempt this in our study due to its pilot nature and a relatively small sample size, this could be a focus of future research, particularly with a larger sample size. A multi-ethnic study using a standardised protocol is needed to find out the optimal cut-off point for GA for detecting IH and DM.

Our study has limitations as well as points of strengths.

The main strength of this study lies in it being the first study in Saudi Arabia to evaluate GA as a new tool for assessing glycaemic status in Saudi adults not previously diagnosed with DM. Additionally, this study compares GA with the currently recommended measures of glycaemia assessment to establish cut-off points for detecting IH and DM. GA has been proposed as offering advantages over other current measures of hyperglycaemia, particularly in the presence of certain medical conditions common among Saudis. The primary limitation of the study is the relatively small sample size. Nevertheless, the sample size was sufficient for this pilot study, providing valuable insights for planning larger future studies. Another limitation is the use of frozen stored samples, which may limit reproducibility in real world setting. However, an earlier study reported that samples frozen at - 70°C and stored for as long as 23 years are suitable for the GA assay (78). Our samples were collected between July 2016 and February 2017, and stored at - 80°C which lends credibility to our methods and findings.

In conclusion, given the high occurrence of DM in Saudi Arabia, there is an urgent need for an effective screening, detection, and monitoring tools suitable for the local population. Additionally, the high rates of conditions affecting erythrocyte integrity pose significant challenges to the reliability of current diagnostic tests used to detect hyperglycaemia in this country. GA emerges as a promising alternative glycaemic test as it is unaffected by medical conditions associated with erythrocyte integrity, thus offering accurate and reliable results essential for early detection of DM and monitoring of its control.

Future research on GA as a diagnostic test for IH and DM should focus on studies with a larger sample size to establish reference values for the diagnosis of IH and DM in the Saudi population and beyond. Longitudinal studies are needed to assess whether GA can serve as a better marker for monitoring DM management and predicting complications associated with hyperglycaemia.

## Data Availability

The original contributions presented in the study are included in the article/supplementary material. Further inquiries can be directed to the corresponding author.
